# “Burden of osteoporotic fractures in primary health care in Catalonia (Spain): a population-based study”

**DOI:** 10.1186/1471-2474-13-79

**Published:** 2012-05-28

**Authors:** Aina Pagès-Castellà, Cristina Carbonell-Abella, Francesc Fina Avilés, Maite Alzamora, Jose Miguel Baena-Díez, Daniel Martínez Laguna, Xavier Nogués, Adolfo Díez-Pérez, Daniel Prieto-Alhambra

**Affiliations:** 1 Institut Català de la Salut, Barcelona, Spain; 2 Universitat Autònoma de Barcelona, Department of Medicine, Barcelona, Spain; 3 Universitat de Barcelona, Department of Medicine, Barcelona, Spain; 4 IDIAP Jordi Gol (Primary Health Care Research Institute), USR Barcelona and USR Metropolitana Nord, Barcelona, Spain; 5 Centre de Salut la Marina, Institut Català de la Salut, Barcelona, Spain; 6 URFOA, IMIM, Internal Medicine Department, Parc de Salut Mar, Barcelona, Spain; 7 RETICEF (Red Temática de Investigación Cooperativa en Envejecimiento y Fragilidad), Instituto Carlos III, Barcelona, Spain

## Abstract

**Background:**

Knowledge on the epidemiology of non-hip fractures in Spain is limited and somewhat outdated. Using computerized primary care records from the SIDIAP database, we derived age and sex-specific fracture incidence rates for the region of Catalonia during the year 2009.

**Methods:**

The SIDIAP database contains quality-checked clinical information from computerized medical records of a representative sample of >5,800,000 patients (80% of the population of Catalonia). We conducted a retrospective cohort study including all patients aged ≥50 years, and followed them from January 1 to December 31, 2009. Major osteoporotic fractures registered in SIDIAP were ascertained using ICD-10 codes and validated by comparing data to hospital admission and patient-reported fractures records. Incidence rates and 95% confidence intervals were calculated.

**Results:**

In total, 2,011,430 subjects were studied (54.6% women). Overall fracture rates were 10.91/1,000 person-years (py) [95%CI 10.89–10.92]: 15.18/1,000 py [15.15–15.21] in women and 5.78/1,000 py [5.76–5.79] in men. The most common fracture among women was wrist/forearm (3.86/1,000 py [3.74–3.98]), while among men it was clinical spine (1.25/1,000 py [1.18–1.33]). All fracture rates increased with age, but varying patterns were observed: while most of the fractures (hip, proximal humerus, clinical spine and pelvis) increased continuously with age, wrist and multiple rib fractures peaked at age 75–80 and then reached a plateau.

**Conclusions:**

Our study provides local estimates of age, sex and site-specific fracture burden in primary health care, which will be helpful for health-care planning and delivery. A proportion of fractures are not reported in primary care records, leading to underestimation of fracture incidence rates in these data.

## Background

Osteoporosis is a common public health problem, due to its association with fragility fractures. In the year 2000, approximately 9 million fractures occurred in the world, of which 1.6 million were hip, 1.7 were wrist/forearm and 1.4 million were clinical spine fractures [1]. The annual costs of all fractures reached about 20 and 30 billion US dollars in the United States and the European Union, respectively [2]. As aging populations increase worldwide, projections estimate that the incidence of hip fractures will rise to 6.3 million in the year 2050 [3].

The epidemiology of osteoporotic fractures in Spain is only partially known: several authors have studied and reported the age, sex and geographical distribution of hip fracture [4-6] and its costs [7] using hospital discharge data, but other fragility fractures which do not usually require hospitalization have been poorly described, due to the lack of reliable data. However, primary health care physicians could be a useful source of information to study the burden of such fractures: indeed, in many countries including Spain, general practitioners play an essential role in the diagnosis and management of osteoporosis [8] and in delivering continuity of care after a major fracture [9]. Consistent with this, they receive continuous information on fracture risk and outcomes [10], either from hospital discharge letters [11], accident and emergency reports, or directly from the patients. Hence, valid data on the epidemiology of non-hospitalized fractures could be obtained using primary care databases in Spain, as has been done in other countries such as the United Kingdom [12,13] or Denmark [14,15].

We therefore estimated age and sex-specific fracture incidence rates as registered in primary care electronic medical records (SIDIAP database) for the population of Catalonia aged 50 years or older during the year 2009. Secondly, we assessed the completeness of the fracture coding in this database, when compared to hospital data (for hip fractures) and to patient reports (for hip, clinical spine and wrist fractures).

## Methods

### Source of data and study population

General practitioners (GPs) play an essential role in the public health care system of Spain, as they are responsible for primary health care, long-term prescriptions and specialist and hospital referrals. The Spanish public health care system covers more than 98% of the population. The data in this study was obtained from the SIDIAP Database, comprised of electronic medical records of a representative sample of patients attended by GPs in Catalonia (North-East Spain), covering a population of more than 5.8 million patients (about 80% of the total of 7.5 million population of Catalonia) from 274 primary care practices with 3,414 participating GPs. The SIDIAP data comprises the clinical and referral events registered by primary care health professionals (GPs and nurses) and administrative staff in electronic medical records, comprehensive demographic information, prescription and corresponding pharmacy invoicing data, specialist referrals, primary care laboratory test results, and hospital admissions and their major outcomes. Health professionals gather this information using ICD-10 codes, and structured forms designed for the collection of variables relevant for primary care clinical management, such as country of origin, sex, age, height, weight, body mass index, tobacco and alcohol use, blood pressure measurements, blood and urine test results. Only GPs who meet quality control standards can participate in the SIDIAP database [16]. Encoding personal and clinic identifiers ensures the confidentiality of the information in the SIDIAP Database. Recent reports have shown the SIDIAP data to be useful for epidemiological research [17].

All patients aged ≥50 years registered in the database in 2009 were eligible for the current study.

### Ascertainment of fractures

Fractures registered in 2009 in the SIDIAP database were identified using medical codes for a list of skeletal sites of fracture (Additional file 1), based on the ICD-10 classification. Fractures considered for these analyses were those defined by Center and Eisman [18] as major fractures based on their associated mortality (hip, clinical spine, pelvic, multiple rib and proximal humerus), and the most prevalent minor osteoporotic fracture in our data (wrist/forearm). We could not tease out high impact fractures, as we had no access to free text contained in medical records for confidentiality reasons.

### Validation of fractures in the SIDIAP database

To assess the completeness and accuracy of the fractures coded in the SIDIAP database, we linked and compared our data to ARTPER data, a population-based prospective cohort study that has been ongoing in 28 primary care centres of Catalonia for the last 4 years [19,20]. The ARTPER study included a random sample of 3,786 individuals aged >49 years, and was powered to estimate the prevalence of peripheral arterial disease in the general population and to study its association with cardiovascular outcomes. Further, a question on the occurrence of hip, spine and wrist/forearm fractures (based on the EPOS study questionnaire [21]) was asked of all ARTPER participants in the 4-year follow-up phone questionnaire in an effort to investigate a potential association between cardiovascular disease and fragility fractures. A total of 3,775 patients (99.7% of the initially recruited) answered the question “Since participating in the baseline survey, have you fractured a bone?”. If the response was “yes”, they were asked to report the date of fracture and to identify which bone(s) they broke, choosing from the following categories: spine, hip/femur, wrist/forearm, and other. Of these reports, 3,402 (90.1%) could be verified and linked to the SIDIAP database. A new dataset with anonymized ID and data on patient-reported (from ARTPER) and physician-registered (in SIDIAP records) fractures was constructed for our analysis of the sensitivity, specificity, and positive and negative predictive value of a SIDIAP code for fracture compared to the ARTPER cohort study. In addition, hip fractures in the SIDIAP database were validated by linkage to the 2009 regional hospital admission database (CMBD), which was considered as a gold standard for comparison. We considered hospitalization for hip fracture only when one of the corresponding ICD-9 codes appeared as the primary diagnosis in the hospital admission database.

### Statistical analyses

Age-specific fracture incidence rates for each fracture site were calculated separately for males and females by dividing the number of patients with a fracture by the total person-years of follow-up and plotting the result against age (incidence estimates are available from the corresponding author). The 95% confidence intervals were estimated using the delta method, as proposed by Kirkwood et al. [22].

For the validation of fracture codes in the SIDIAP database, crosstabs were used, with rows indicating SIDIAP fracture (yes/no) and columns indicating either ARTPER fracture (yes/no) or CMBD fracture (yes/no). Specificity, sensitivity and predictive values were estimated for each of the fracture sites studied.

All these analyses were carried out using Microsoft Excel 2008 for Mac, SPSS for Mac version 18.0 and R for Mac version 2.9.1.

### Ethics

SIDIAP provided purely observational data for this study. It obtained approval from the SIDIAP Scientific Committee, responsible for reviewing protocols for scientific quality. The ARTPER cohort study was approved by the local Ethics Committee (IDIAP Jordi Gol i Gurina). Informed consent was obtained from all the participants, and the recommendations of the World Medical Association Declaration of Helsinki were followed throughout the study.

## Results

**Figure 1:**
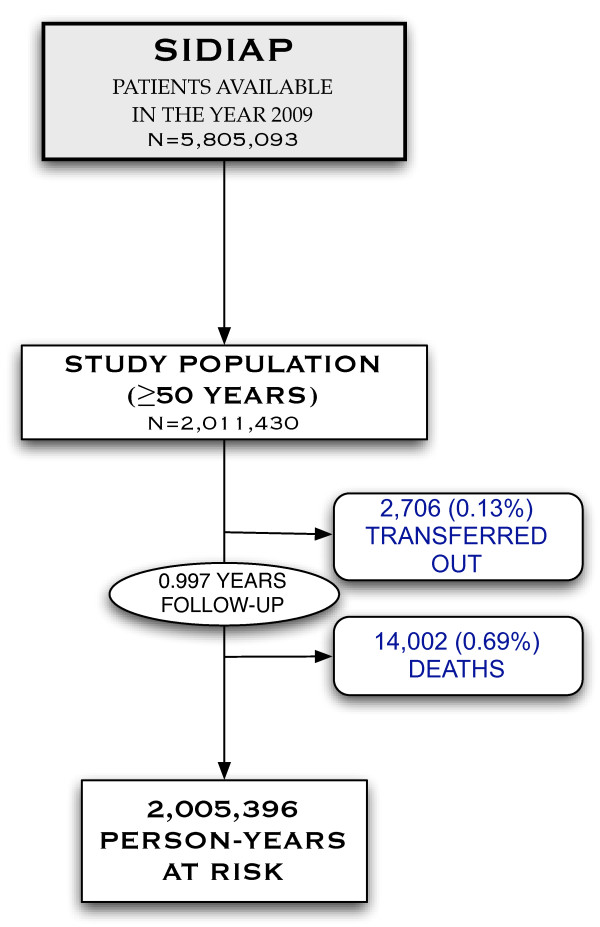
Population flow-chart.

The SIDIAP database included information on 5,805,093 people in the year 2009, of whom 2,011,430 were 50 years or older, and therefore eligible for this study. Of these, 1,098,386 (54.6%) were women, and the mean (standard deviation) age of the study population was 66.9 (12.1) years. Study participants were then followed up for a median of 0.997 years (interquartile range 0.996 to 0.999), with only 0.13% lost to follow-up (Figure 1).

**Figure 2:**
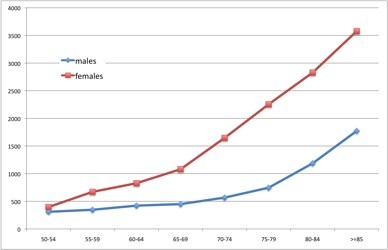
Overall osteoporotic fractures: age and sex-specific incidence rates (per 100,000 person-years at risk).

**Figure 3:**
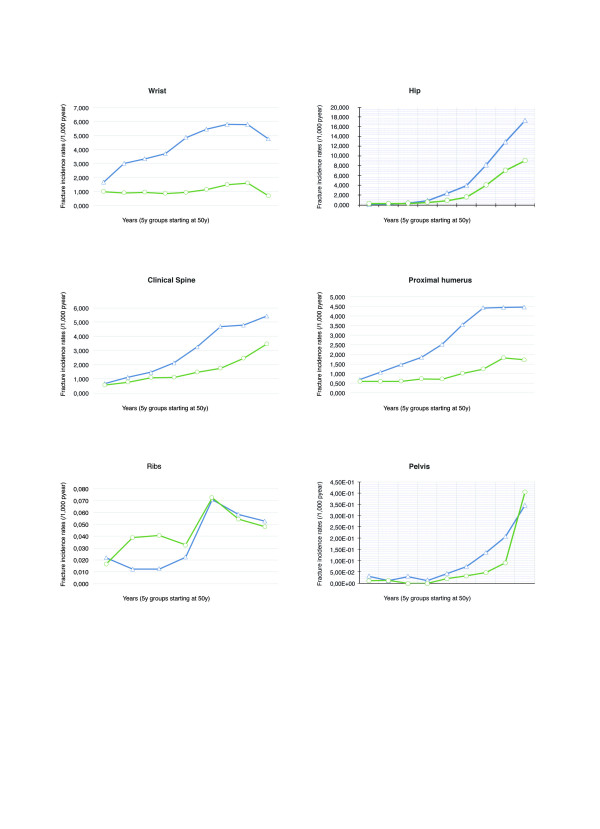
Age and sex-specific incidence rates by fragility-fracture skeletal sites studied.

Overall and site-specific fracture incidence rates were estimated for the full study population and by sex (Table 1). Among women, the overall fracture incidence rate was 15.18/1,000 person-years-at-risk (pyar) [95% CI 15.15 to 15.21], and among men 5.78/1,000 pyar [5.76 to 5.79]. The most common fragility fracture seen in women was wrist/forearm, with an incidence of 3.86/1,000 pyar [3.74 to 3.98], followed by hip fracture, with an incidence rate of 3.08/1,000 pyar [2.97 to 3.18]. In contrast, the most common fracture in men was clinical spine (1.25/1,000 pyar [1.18 to 1.33]), followed by hip fracture (1.23/1,000 pyar [1.16–1.30]). As expected, overall age-specific fracture rates increased with age in both males and females (Figure 2). Among women, rates continued to increase through later adult life to the age of 85 years and beyond, with an incidence rate of 35.77/1,000 pyar [34.60 to 36.97]. In males, the highest overall fracture rate was also seen in participants aged ≥85 years, with an estimated incidence of 17.72/1,000 pyar [16.54 to 18.99]. Age- and sex-specific fracture rates for each of the skeletal sites studied are shown in Figure 3.

**Table 1:** Sex- and site-specific fracture incidence rates [and 95% Confidence Intervals] per 1,000 person-years at risk in the SIDIAP population ≥50 years old

**Skeletal site**	**Women**	**Men**	**Total**
**Overall**	15.18 [15.15–15.21]	5.78 [5.76–5.79]	10.91 [10.89–10.92]
**Hip**	3.08 [2.97–3.18]	1.23 [1.16–1.30]	2.23 [2.16–2.30]
**Wrist/Forearm**	3.86 [3.74–3.98]	1.03 [0.97–1.10]	2.56 [2.49–2.63]
**Clinical spine**	2.59 [2.49–2.69]	1.25 [1.18–1.33]	1.98 [1.91–2.04]
**Proximal humerus**	2.19 [2.11–2.29]	0.78 [0.73–0.84]	1.55 [1.50–1.61]
**Multiple rib**	0.03 [0.02–0.05]	0.04 [0.03–0.05]	0.04 [0.03–0.05]
**Pelvis**	0.06 [0.05–0.08]	0.02 [0.01–0.03]	0.04 [0.03–0.05]

Regarding the validity of fracture coding in the SIDIAP database, at year 4 of follow-up the 3,402 participants in the ARTPER study reported 57 wrist, 18 hip and 6 clinical spine fractures (Table 2). Corresponding sensitivity, specificity, and positive and negative predictive values were, respectively: 56.1%, 99.8%, 82.1% and 99.3% for wrist/forearm; 66.7%, 99.9%, 92.3% and 99.8% for hip; and 50%, 99.9%, 37.5% and 99.9% for clinical spine fractures. In the comparison between SIDIAP and hospital discharge data, 1,686 hip fractures were observed among SIDIAP participants, and 2,063 in the data corresponding to regional hospital admission database (CMBD) for hip fracture (Table 2). Corresponding values were: 60.1% sensitivity, 99.9% specificity, 70.8% positive predictive value and 99.9% negative predictive value.

**Table 2:** Fractures registered in the SIDIAP database, in the ARTPER cohort study and in the CMBD (hospital admission) database

**SKELETAL SITE**	**Reported in ARTPER**	**Unreported in ARTPER**
WRIST/FOREARM		
Recorded in SIDIAP	32 (82.1%)	7 (17.9%)
Not recorded in SIDIAP	25 (0.7%)	3,338 (99.3%)
CLINICAL SPINE		
Recorded in SIDIAP	3 (37.5%)	5 (62.5%)
Not recorded in SIDIAP	3 (0.1%)	3,391 (99.9%)
HIP		
Recorded in SIDIAP	12 (92.3%)	1 (7.7%)
Not recorded in SIDIAP	6 (0.2%)	3,383 (99.8%)
HIP IN HOSPITAL DATA	**Recorded in CMBD**	**Not recorded in CMBD**
Recorded in SIDIAP	1,194 (70.8%)	492 (29.2%)
Not recorded in SIDIAP	869 (0.01%)	1,119,624 (99.9%)

## Discussion

### Main results

The current study estimates for the first time the burden of several osteoporotic fractures in both men and women attending primary health care centres in Catalonia. According to our data, fracture incidence rates are higher for women for almost all of the skeletal sites and age groups studied. Fracture incidence rises continuously with increasing age for all the fracture sites studied except for wrist/forearm and multiple rib fracture: for these, we observed a peak incidence at the age of 75–80 years, and either a plateau or a decline in older ages. Whilst the most frequent fracture site for women is the wrist/forearm, it is the spine that is most common for men.

In addition, we have shown that the SIDIAP database contains valid clinical data for the study of the epidemiology of osteoporotic fractures. Fracture coding in SIDIAP has low sensitivity when compared to conventional cohort studies (between 50% and 70% depending on fracture site), consistent with some degree of under-reporting of fracture events in primary care. Conversely, the data in SIDIAP are highly specific (always >99%), and thus highly reliable for a confirmable fracture. All these values are, as expected, higher for hip than for wrist or clinical spine fracture.

### Interpretation

Although Spain has been identified by Kanis et al. [23] as a country with a moderate risk for fracture, within the Spanish territory Catalonia has been described as the region with the highest risk of hip fracture: Alvarez-Nebreda et al. [4] used hospital discharge data to study the epidemiology of hip fracture in the different Spanish regions, and estimated a risk of such fracture of 8.46/1,000 pyar among the Catalan female population and 3.50/1,000 pyar in men in the period 2000–2002. Hernandez et al. [5] studied the trends of hip fracture in the region of Cantabria for a 14-year period (1988–2002) and found much lower rates, with estimated incidences of 2.77/1,000 pyar and 1.00/1,000 pyar for women and men, respectively. A similar study was carried out by Naves-Diaz M et al. [24], who estimated the risk of hip fracture in Asturias, demonstrating incidence rates of 3.25/1,000 pyar for women and 1.40/1,000 pyar for males living in the same area. However, only one study to date has studied the burden of osteoporotic fractures in Spain’s Primary Health Care settings: the ECOSAP study [25] enrolled only women, including some from Catalonia, and showed a hip fracture incidence of 3.60/1,000 pyar. There are at least two explanations for the disparity between these results and ours: 1. the under-reporting of osteoporotic fractures in primary care records, which we have shown are highly specific but have low sensitivity when compared to cohort studies and hospital discharge data, and 2. the age of the study participants, which averaged 5 to 13 years older in these studies than in ours. Similarly, when we compare our estimates to those obtained in these same studies for the incidence of other fractures, such as wrist/forearm, their results are always higher (7.93/1,000 pyar in the female population assessed in Asturias [24], and 8.87/1,000 in ECOSAP [25]), probably for the same reasons described above. Clinical spine fractures were not reported by any of these authors and proximal humerus, rib and pelvis fractures were only studied in ECOSAP, which did not include men. In an international context, Van Staa et al. [26] carried out a very similar study to ours in an effort to describe the epidemiology of fractures in England and Wales using data from general practice records (GPRD database). In that study, the pattern of age and sex-specific fracture incidence rates for the different skeletal sites assessed here were very similar to ours. Further, the most common fractures they found among women support our findings. However, this comparison might be limited by different characteristics of the study population (older than 20 in the GPRD study and ≥50 years in ours) and other methodological differences. These same authors studied the validity of fracture coding within the British GPRD database [12] and found slightly higher values of concordance both with GP questionnaires and with discharge summary than we report here.

Causes of under-coding of fractures in primary care medical records can vary, but may include non-reporting of high-energy trauma fractures (e.g. traffic collisions leading to fractures attended directly in hospital settings) and use of private health care services.

### Strengths and limitations

The main limitations of our data are the lack of individual validation for each fracture identified, as well as on the methods used for fracture diagnoses and on the nature of the impact that caused these fractures (high impact trauma versus fragility fractures). However, hip fractures were validated using hospital admission data and a sample of other fractures (wrist/forearm and clinical spine) were confirmed by patient-reported data within a prospective cohort study including almost 3,500 patients. On the other hand, this is the first population-based study to describe the burden of osteoporotic fractures in the Primary Health Care system of Catalonia. In addition, the diversity of skeletal sites validated in this study is much greater than in any previous study in Spain.

## Conclusions

We have demonstrated that the overall burden of osteoporotic fractures in the Catalan Primary Health Care setting is remarkable, with an incidence of almost 11/1,000 pyar. In a representative primary care practice, where each GP has on average 1,500 patients and about 40% of them are aged 50 years or older, each physician would be seeing 6 to 7 new osteoporotic fractures per year. In general terms, because older patients and women are at a higher risk of suffering fragility fractures, practices covering aged populations could potentially be seeing a much higher number of fractures.

We have also shown that medical records from the SIDIAP database are a valuable source of data for the investigation of the epidemiology of osteoporotic fractures, with some under-reporting (particularly present for clinical spine fractures) but high specificity and predictive values when compared to hospital and cohort data.

## Abbreviation

Prox Hum: Proximal humerus.

## Competing interests

All the authors report no conflicts of interest regarding this piece of work.

## Authors’ contributions

APG, CCA, XN, ADP and DPA contributed to study design; APG, CCA, FFA, MA, JMBD, DML and DPA carried out the acquisition of data; APG, CCA and DPA drafted the manuscript; all the authors had access to the data and critically reviewed the manuscript before submission. All authors read and approved the final manuscript.

## Funding

This study has received partial funding from the Ministerio de Sanidad, Política Social e Igualdad (Government of Spain), through the Ayudas a la Investigación Independiente 2010 scheme: the CUTE-REINA-BIS Study; reference EC10-080.

Daniel Prieto-Alhambra receives support from the IDIAP Jordi Gol and Institut Catala de la Salut (*“4a Convocatòria d’una estada a una Unitat de Recerca de l’IMIM o de l’ASPB”*). The Internal Medicine Department and the URFOA IMIM receive support from the RETICEF (Red Temática de Investigación Cooperativa en Envejecimiento y Fragilidad, Instituto Carlos III, Government of Spain).

## Pre-publication history

The pre-publication history for this paper can be accessed here:

http://www.biomedcentral.com/1471-2474/13/79/prepub

## Supplementary Material

Additional file 1 ICD-10 Codes used to identify fractures in the SIDIAP database. Click here for file
