# Supplementary material for: “Burden of osteoporotic fractures in primary health care in Catalonia (Spain): a population-based study”
Source: BMC Musculoskelet Disord. 2012 May 28;13:79. doi: 10.1186/1471-2474-13-79 (PMC3489545; doi:10.1186/1471-2474-13-79)
Supplement: Additional file 1 — ICD-10 Codes used to identify fractures in the SIDIAP database. [file 1471-2474-13-79-S1.doc]

**APPENDIX 1**. ICD-10 Codes used to identify fractures in the SIDIAP database.

| **ICD-10 CODE** | **DESCRIPTION** |
| --- | --- |
| S72 | FRACTURE OF FEMUR |
| S72.0 | FRACTURE OF NECK OF FEMUR |
| S72.1 | PERTROCHANTERIC FRACTURE |
| S72.9 | FRACTURE OF FEMUR, PART UNSPECIFIED |
| M48.4 | FATIGUE FRACTURE OF VERTEBRA |
| S12 | FRACTURE OF NECK |
| S12.0 | FRACTURE OF FIRST CERVICAL VERTEBRA |
| S12.1 | FRACTURE OF SECOND CERVICAL VERTEBRA |
| S12.2 | FRACTURE OF OTHER SPECIFIED CERVICAL VERTEBRA |
| S12.7 | MULTIPLE FRACTURES OF CERVICAL SPINE |
| S12.8 | FRACTURE OF OTHER PARTS OF NECK |
| S12.9 | FRACTURE OF NECK, PART UNSPECIFIED |
| S22.0 | FRACTURE OF THORACIC VERTEBRA |
| S22.1 | MULTIPLE FRACTURES OF THORACIC SPINE |
| S32 | FRACTURE OF LUMBAR SPINE AND PELVIS |
| S32.0 | FRACTURE OF LUMBAR VERTEBRA |
| S32.1 | FRACTURE OF SACRUM |
| S32.2 | FRACTURE OF COCCYX |
| S32.7 | MULTIPLE FRACTURES OF LUMBAR SPINE AND PELVIS |
| S32.8 | FRACTURE OF OTHER AND UNSPECIFIED PARTS OF LUMBAR SPINE AND PELVIS |
| T08 | FRACTURE OF SPINE, LEVEL UNSPECIFIED |
| S22.4 | MULTIPLE FRACTURES OF RIBS |
| S22.5 | FLAIL CHEST |
| S42 | FRACTURE OF SHOULDER AND UPPER ARM |
| S42.2 | FRACTURE OF UPPER END OF HUMERUS |
| S52 | FRACTURE OF FOREARM |
| S52.2 | FRACTURE OF SHAFT OF ULNA |
| S52.3 | FRACTURE OF SHAFT OF RADIUS |
| S52.4 | FRACTURE OF SHAFTS OF BOTH ULNA AND RADIUS |
| S52.5 | FRACTURE OF LOWER END OF RADIUS |
| S52.6 | FRACTURE OF LOWER END OF BOTH ULNA AND RADIUS |
| S52.7 | MULTIPLE FRACTURES OF FOREARM |
| S52.8 | FRACTURE OF OTHER PARTS OF FOREARM |
| S52.9 | FRACTURE OF FOREARM, PART UNSPECIFIED |
| S62 | FRACTURE AT WRIST AND HAND LEVEL |
| S22 | FRACTURE OF RIB(S), STERNUM AND THORACIC SPINE |
| S22.2 | FRACTURE OF STERNUM |
| S22.3 | FRACTURE OF RIB |
| S42.3 | FRACTURE OF SHAFT OF HUMERUS |
| S42.4 | FRACTURE OF LOWER END OF HUMERUS |
| S62.8 | FRACTURE OF OTHER AND UNSPECIFIED PARTS OF WRIST AND HAND |
| S72.7 | MULTIPLE FRACTURES OF FEMUR |
| S72.8 | FRACTURES OF OTHER PARTS OF FEMUR |
| S32.3 | FRACTURE OF ILIUM |
| S32.4 | FRACTURE OF ACETABULUM |
| S32.5 | FRACTURE OF PUBIS |
